# Social science perspectives on drivers of and responses to global climate change

**DOI:** 10.1002/wcc.554

**Published:** 2018-09-09

**Authors:** Andrew K. Jorgenson, Shirley Fiske, Klaus Hubacek, Jia Li, Tom McGovern, Torben Rick, Juliet B. Schor, William Solecki, Richard York, Ariela Zycherman

**Affiliations:** ^1^ Department of Sociology Boston College Chestnut Hill Massachusetts; ^2^ Department of Anthropology University of Maryland College Park Maryland; ^3^ Department of Geographical Sciences University of Maryland College Park Maryland; ^4^ U.S. Environmental Protection Agency Washington DC; ^5^ Department of Anthropology Hunter College‐CUNY New York New York; ^6^ Department of Anthropology, National Museum of Natural History Smithsonian Institution Washington DC; ^7^ Department of Geography Hunter College‐CUNY New York New York; ^8^ Department of Sociology University of Oregon Eugene Oregon; ^9^ USDA–National Institute of Food and Agriculture Washington DC

**Keywords:** adaptation, anthropology, archeology, climate change, geography, greenhouse gas emissions, mitigation, social science, sociology, sustainability

## Abstract

This article provides a review of recent anthropological, archeological, geographical, and sociological research on anthropogenic drivers of climate change, with a particular focus on drivers of carbon emissions, mitigation and adaptation. The four disciplines emphasize cultural, economic, geographic, historical, political, and social‐structural factors to be important drivers of and responses to climate change. Each of these disciplines has unique perspectives and makes noteworthy contributions to our shared understanding of anthropogenic drivers, but they also complement one another and contribute to integrated, multidisciplinary frameworks. The article begins with discussions of research on temporal dimensions of human drivers of carbon emissions, highlighting interactions between long‐term and near‐term drivers. Next, descriptions of the disciplines' contributions to the understanding of mitigation and adaptation are provided. It concludes with a summary of key lessons offered by the four disciplines as well as suggestions for future research.

This article is categorized under:

Climate Economics > Economics and Climate Change

## 1 INTRODUCTION

The drivers of climate change are explored in a wide range of scientific and climate assessment literatures. “Anthropogenic” drivers refer to the human actions that cause climate change and the societal factors that shape and condition those actions (Rosa & Dietz, 2012). More broadly, human driving forces, in this context, are the range of characteristics of societies that have substantial influence on the global climate (Dietz, Rosa, & York, 2010; Rosa, Rudel, York, Jorgenson, & Dietz, 2015).

Emissions and atmospheric concentrations of long‐lived greenhouse gases (GHG), especially carbon emissions, have increased dramatically since the pre‐industrial period. That increase is due primarily to human activities associated with fossil‐fuel use and agriculture, while other land‐use changes, such as deforestation, provide significant but smaller contributions (IPCC, 2007, 2014a; USGCRP, 2017). The scale of these emissions has been an important component in the designation of a new geological era in which human activity is a primary driver—the Anthropocene.

This article reviews recent anthropological, archeological, geographical, and sociological perspectives on anthropogenic drivers of climate change, with a particular focus on drivers of carbon emissions. Attention is also given to some of the ways in which these social science disciplines contribute to research on mitigation and adaptation. As a whole, the four disciplines emphasize cultural, economic, geographic, historical, political, and social‐structural factors to be important drivers of and responses to climate change. While each of these disciplines has unique perspectives and makes noteworthy contributions to our shared understanding of the human dimensions of climate change, they also complement one another and contribute to integrated, multidisciplinary frameworks (Dietz, Sovacool, & Stern, 2016; Stern, Sovacool, & Dietz, 2016).

While far from an exhaustive review of what the four disciplines have to offer, the article covers important contributions from these disciplines that inform knowledge of drivers and responses to climate change. It begins with discussions of temporal dimensions, identifying and presenting interactions between long‐term and near‐term human drivers of carbon emissions. Next, descriptions of the disciplines' contributions to the understanding of mitigation and adaptation are provided. The conclusion includes a summary of key lessons offered by the four disciplines as well as suggestions for future research.

## 2 DRIVERS AND THEIR INTERACTIONS OVER TIME

The long‐term and near‐term drivers of climate change continuously interact (Dietz, 2017). Among the anthropogenic drivers of carbon emissions that anthropology, archeology, geography, and sociology investigate are economic systems, including growth, cycles, consumption, and trade; power, social stratification, and inequality; population growth and demographic shifts; technology; infrastructure; and land‐use change and land transformation. “Long” and “near” terms are often defined differently within and between disciplines. “Long‐term” may be applied to several decades, one or more centuries, or even longer periods, while “near‐term” may refer to a period shorter than a year.

### 2.1 Social dimensions of economic systems

This section focuses on the economic activities and trends that lead to increased carbon emissions. Section 2.1.1 considers the relationships between national economic growth and local‐level processes, and the impacts these factors have on emissions. Section 2.1.2 focuses primarily on the broader impacts of collective and individual economic practices, and also provides a brief summary of recent research on nations' militaries.

#### 2.1.1 Economic growth and cycles

One of the major drivers of climate change is economic growth, which includes long‐term and near‐term factors that influence the timing and extent of the driver's impact. Sociological research has employed longitudinal modeling techniques and statistical interactions to assess the potentially changing effect of economic development on national‐level carbon emissions (Jorgenson & Clark, 2012; Knight & Schor, 2014; Longhofer & Jorgenson, 2017; Thombs, 2018a). This body of research provides a sociological approach to analyses of a potential decoupling of gross domestic product (GDP) and emissions (OECD, 2002), and commonly focuses on testing hypotheses derived from social theories, particularly ecological modernization theory and treadmill of production theory.

Briefly, ecological modernization theory suggests that processes of modernization result in added reflexivity throughout the socioeconomic system (Mol, 2003; Mol, Spaargaren, & Sonnenfeld, 2014). Technological development and environmental consciousness, both of which go hand‐in‐hand with economic development, are seen as key components of modernization via the greening of industrial production processes, including reduced fossil fuel consumption, while also leading to more sustainable forms of consumption. In contrast, treadmill of production theory argues that since market economies are predicated on increasing profits through expansion, energy consumption and forms of pollution continually expand while overall environmental conditions deteriorate (Gould, Pellow, & Schnaiberg, 2008; Schnaiberg, 1980). Thus, economic development involves increases in resource use and waste generated from the various stages in production processes, including increased fossil fuel consumption and resultant carbon emissions.

In the first study in sociology that takes such an approach, Jorgenson and Clark (2012) use interactions between GDP per capita and time in longitudinal models of anthropogenic carbon emissions for samples of developed and developing countries covering the 1960–2005 period. Three measures of national carbon emissions are analyzed: total emissions, per capita emissions, and emissions per unit of GDP. The results indicate a strong relationship between per capita emissions and GDP per capita in developed nations that is stable in magnitude through time. For developing countries they find that the association between emissions and GDP per capita increases in magnitude through time, the opposite of decoupling, but still remains smaller in magnitude than the relationship between per capita emissions and GDP in developed countries. For total emissions, the estimated effect of GDP per capita decreased in magnitude over time in developed countries, providing some evidence of a decoupling for such nations, while for developing countries the results indicate a stable effect of GDP per capita on total emissions through time. The analysis of emissions per unit of GDP suggests a slight decoupling for the sample of developed nations, while the findings for the sample of developing countries are inconclusive. In a more recent longitudinal study that extends the temporal scope of the analysis to 2010, Thombs (2018a) replicates the findings of Jorgenson and Clark (2012) across all three measures of emissions. Overall, this body of research on decoupling provides mixed support for ecological modernization theory and treadmill of production theory, and suggests that both frameworks could benefit from further consideration of how the global organization of production and the structure of international trade influence the relationship between carbon emissions and economic development.

Similar modeling techniques that include interactions between time and measures of GDP per capita have been used in studies of the effect of economic growth on the “carbon intensity of human well‐being” (CIWB)—a ratio between per capita carbon emissions and a measure of human well‐being—for samples of nations in the Americas, Europe, Oceania, Asia, and Africa (Jorgenson, 2014). Findings suggest that the effect of GDP per capita on CIWB is relatively large, positive, and stable in magnitude through time for nations in North America, Europe, and Oceania, and has increased in magnitude through time for nations in the other regional samples (Jorgenson, 2014; see also Dietz, 2015; Dietz, Rosa, & York, 2009, Dietz, Rosa, & York, 2012; Mayer, 2017; Mazur & Rosa, 1974).

Recent sociological analyses of national carbon emissions have also highlighted notable temporal differences in the effects of urbanization across regions, which is tightly connected to processes of economic growth (Jorgenson, Auerbach, & Clark, 2014). For nations in Asia, the estimated effect of urbanization (measured as the percent of the nation's population residing in urban areas) on emissions has increased in magnitude through time, while for nations in Latin America, the estimated effect of urbanization has fluctuated slightly through time, but continues to be moderate in magnitude. For the wealthier nations in North America, Europe, and Oceania, the effect of urbanization on emissions is larger than for nations in other regions, but gradually decreased in the 1980s and 1990s, followed by a moderate increase in the early 2000s. Related research shows that in developing nations with a larger urban slum prevalence, the overall effect of urbanization on carbon emissions is suppressed to some extent, given that households in urban slums are structurally disadvantaged and generally consume less fossil fuel energy and other carbon‐intensive goods (Givens, 2015; Jorgenson, Rice, & Clark, 2010; McGee, Ergas, Greiner, & Clement, 2017), which highlights socioeconomic heterogeneity and structural inequities within urban contexts (Elliott & Clement, 2014).

Drivers that show the most noticeable effects over short periods can demonstrate how economic cycles increase or decrease emissions. In the United States, for example, carbon emissions from the burning of fossil fuels declined between 2007 and 2013. According to research by geographers, the economic recession was more important than the substitution of natural gas for coal in the power sector for explaining the decline (Feng, Davis, Sun, & Hubacek, 2015). Relevant factors were a reduction in overall economic activity, changes in the production of industrial goods because private sector enterprises were less willing to invest in capital formation, and a moderate increase in the use of renewable energy relative to fossil fuel energy (Feng, Davis, Sun, & Hubacek, 2016). Historically, technical progress has only partially compensated for additional emissions from economic growth in both national and international contexts (Jackson et al., 2016; Peters, Weber, Guan, & Hubacek, 2007; Yao, Feng, & Hubacek, 2015).

The collapse of the Soviet Union also led to declines in GHG emissions. During the 1990s, the former Soviet republics experienced demographic and economic decline along with de‐urbanization, all of which influenced carbon emissions. Studying these trends, York (2008), a sociologist, finds not only that those reversals led to reductions in carbon emissions but also that the events in the former Soviet republics provided a clear example of dramatic carbon emissions reductions. The related infrastructural momentum (the energy capacity and demand stemming from durable infrastructure like roads, pipelines, and factories, that persist even in economic downturns), however, meant that former Soviet republics still had higher emissions than other low to moderate income nations that had not industrialized so intensively (York, 2008).

Although the economic collapse in former Soviet republics obviously does not present a desirable model to reduce emissions, the Soviet example shows that once energy intensive infrastructure is in place, it generates some degree of energy consumption that is hard to suppress. Subsequent research in sociology has established a general pattern across nations where economic decline does not reduce carbon emissions and energy use as much as economic growth spurs them, which once again highlights the degree to which energy demand can be difficult to curtail once industrial infrastructure has been developed (York, 2012a; York & Light, 2017).

Sociologists have also analyzed differences in how components of GDP contribute to emissions. For example, a series of cross‐sectional (Schor, 2005) and longitudinal studies (Fitzgerald, Jorgenson, & Clark, 2015; Fitzgerald, Schor, & Jorgenson, 2018; Knight, Rosa, & Schor, 2013) show that working hours are strongly associated with carbon emissions, whether considering just developed countries, both developing and developed countries, or within the United States, across states. The strongest relationship is what has been called the “scale effect:” the number of hours worked is associated with the size of aggregate output. Countries that have reduced average hours, such as Northern European countries, are lower emitters, ceteris paribus. The second, smaller channel of influence, (the “composition effect”) occurs via household decision‐making: holding income constant, households with less free time engage in more carbon‐intensive consumption. This hypothesized effect has not been measured directly, but is evident in economy‐wide analyses.

#### 2.1.2 Consumption

As consumption is frequently the largest component of output, social scientists have studied its role in GHG emissions. Thus, the rise of consumer society or consumer culture, in which human beings increasingly practice a consumer‐oriented way of life, can be understood as a driver of emissions (Baudrillard, 2017). Income, infrastructure, social organization, and culture all affect expenditure patterns and investment and in turn have direct effects on climate change. Higher income and wealth generally lead to higher energy consumption and carbon emissions. And according to research in geography, the urbanization of populations, particularly in low and moderate income settings, is also associated with the development of high resource‐consuming lifestyles (Leichenko & Solecki, 2005).

Carbon disparity across nations changes with increasing income. Research in geography by Hubacek et al. (2017a) shows that when countries are ranked from lowest to highest income, substantial variation becomes evident among lower‐income countries: there is a declining disparity of carbon footprints within a country as income increases. The disparity in carbon footprints declines as countries become richer, but the average carbon footprint increases along with income. They also find for all countries that the carbon footprint grows with increasing income even though carbon intensity tends to decline, that is, more lower carbon consumption expenditures (such as a larger share of healthcare or education) are added into the consumption mix as income rises.

Anthropologically and sociologically, understanding consumption as a driver takes into account cultural and social contexts (Dietz, Stern, & Weber, 2013), such as through examining status consumption and status competition (Ehrhardt‐Martinez & Schor, 2015; Wilk, 2010). Status seeking contributes to emissions as it leads people to purchase carbon‐intensive consumer goods and services, such as large homes, large vehicles, frequent vacations, and other luxuries, which have tended to serve as status markers on account of their social visibility (Schor, 1998). Consumption patterns are also capable of helping to reduce emissions, however, when green products such as hybrid vehicles or solar roof installations become high‐status indicators (Griskevicus, Tybur, & Van Den Bergh, 2010).

Consumer practices are important as well. An increase in energy‐intensive practices, such as greater use of heating and cooling or a shift to daily showering, tends to increase emissions, but modifying these practices or adopting others, such as choosing public transportation over driving, can reduce emissions (Dietz, Gardner, Gilligan, Stern, & Vandenbergh, 2009; Ehrhardt‐Martinez & Schor, 2015; Shove, Pantzar, & Watson, 2012). Choosing green energy options, such as rooftop solar photovoltaic systems, has been shown to have a strong spatial pattern of adoption leading to the conclusion that “peer effects” can be a strong force in consumer choices. Adoption often occurs among neighboring residences, irrespective of economic class and political party (Graziano & Gillingham, 2015).

The “lifestyle” concept is also useful in analyzing carbon emissions. The ways in which people live and consume are reflected in the consumption patterns of societal groups with different socioeconomic characteristics, such as identity, education, employment, or family status (Baiocchi, Minx, & Hubacek, 2010). Housing is one significant aspect of lifestyle‐related choices (Huddart Kennedy, Krahn, & Krogman, 2014). Suburbanites, especially in more affluent nations, generally purchase large, capacious homes with substantial heating and cooling requirements. Commuting distance and access to public transportation, recreation areas, city centers, public services, and shops are other important neighborhood‐specific, lifestyle‐associated determinants of carbon emissions (Baiocchi et al., 2010). Drivers for different lifestyle groups have been assessed by geographers at fine spatial scales using big data (Hubacek et al., 2016). Geo‐demographics uses a large set of spatially specific variables of characteristics that account for household context as it contributes to emission patterns. The key determinants of lifestyle‐related emissions, as identified through this type of analysis, could also impede change and emissions reduction.

A growing area of environmental social science research on consumption focuses instead on nations' militaries. For example, recent longitudinal analyses within sociology link higher levels of national‐level energy consumption and carbon emissions to the relative size (measured as military participation rate) and capital intensity (measured as military expenditures as % GDP and military expenditures per soldier) of nation's militaries, a finding which holds for both developed and developing nations (Bradford & Stoner, 2017; Clark, Jorgenson, & Kentor, 2010; Jorgenson, Clark, & Kentor, 2010). A network of military bases encompasses the globe, requiring the consumption of a vast amount of resources—including fossil fuels—to staff, operate, and transport equipment and personnel between destinations (Gould, 2007; Hooks and Smith 2004, 2005, 2012). Common military equipment, such as planes, ships, helicopters, tanks, and vehicles requires the consumption of large amounts of energy. For example, 1 hr of operation of a nonnuclear aircraft carrier consumes 21,300 L (over 5,621 gal) of fossil fuel; large, high‐tech military helicopters burn five gallons of fuel for every mile that they travel; and fighter planes, such as the F‐15 and F‐16, consume between 1,500 and 1,700 gal of fuel per hour. If their afterburners are used, up to 14,400 gal are exhausted per hour (Clark & Jorgenson, 2012). This body of research on the environmental impacts of nations' militaries is also relevant for approaches to international inequality and ecologically unequal exchange, which are discussed below.

### 2.2 Power, social stratification, and inequality

Interactions among power, social stratification, and inequality—whether international, regional, national, or subnational—all affect emissions and climate change. Along with the United States, the highest‐emission nations include China, India, and Brazil. Who wields power in those nations? The answer to that question has national and international policy implications that not only affect global changes but also influences how local populations experience and contribute to growth in carbon emissions and climate change.

Theoretical perspectives from social science that address questions of power and inequality include political economy and political ecology, as well as ideas about state action and individual choice and behavior. Research in geography and sociology indicates that recent decades have seen increased global outsourcing, through manufacturing or extraction, of pollution from wealthier countries to poorer ones (Jorgenson, 2007; Jorgenson, Dick, & Mahutga, 2007; Prell & Feng, 2016) and among regions within a nation (Collins, Munoz, & JaJa, 2016; Feng et al., 2013; Williams, 2001). Poor regions often provide inputs and labor for global production networks, and are the locations of the stages of energy‐intensive production that contribute heavily to pollution, including carbon emissions from the burning of fossil fuels (Feng et al., 2013; Grimes & Kentor, 2003; Prell, Feng, Sun, Geores, & Hubacek, 2014).

In terms of benefits and costs along global supply chains, the current structure of those chains tends to reify international inequalities in the world system (Chase‐Dunn & Grimes, 1995). Larger shares of value added, in comparison to shares of pollution, are generally prompted within more‐developed countries, while less‐developed countries experience more environmental destruction and associated health impacts per unit of value added for their contribution to global supply chains (Burns, Davis, & Kick, 1997; Greiner & McGee, 2018; Prell et al., 2014; Prell & Feng, 2016). While China, as of this writing, is experiencing the greatest negative effects, other nations and regions play similar roles. Bangladesh, Cambodia, and India are leading producers of textiles, and Laos, Myanmar, and several nations in Africa have many sites of “land grabs,” the buying or leasing of land for export production on terms unfavorable to local people, with consequences for the environment and climate change, including deforestation (Marselis, Feng, Liu, Teodoro, & Hubacek, 2017).

Global inequalities can also be considered from the perspective of households, rather than nations. Globally, households with incomes in the top 10% are responsible for 36% of carbon emissions, while those in the bottom 50% are responsible for only 15% of emissions. The average annual carbon footprint of global elites is about 14 times that of the lowest income group. In 2010, these footprints ranged from 26.3 tons for the highest global income category to 1.9 tons for the lowest (Hubacek et al., 2017b).

Recent studies in sociology and geography have looked at domestic inequality as a driver of emissions, finding that domestic inequality of both income and wealth are positively associated with carbon emissions, especially via concentration of income and wealth at the top of the distribution. These associations are observed within more economically developed nations, such as the United States (Jorgenson, Schor, & Huang, 2017; Jorgenson, Schor, Knight, & Huang, 2016; Knight, Schor, & Jorgenson, 2017) and in developing nations as well (Hubacek et al., 2017a).

A number of factors account for the positive associations between emissions and income inequality and wealth inequality. Higher‐income and wealthier groups tend to consume more goods and services as they engage in Veblenian status‐consumption (Veblen, 1934) or consumption competition (Schor, 1998). These dynamics lead households to increase their spending to keep up with the visible lifestyles of higher‐income, wealthier households, which in recent decades has entailed consumption of energy‐intensive luxuries such as multiple homes and private planes. The wealthy are also owners of polluting firms and energy producing enterprises. To protect these assets, they are more likely to use their economic resources to gain political power, which they use to dominate the policy environment (Downey, 2015; Jorgenson, Schor, & Huang, 2017; Knight et al., 2017; Prell, Sun, Feng, & Myroniuk, 2015). An additional pathway is that income inequality has been shown to have a positive association with working hours (Bowles & Park, 2005), and recent sociological research, reviewed above, has shown that increased working hours are drivers of energy consumption and carbon emissions (e.g., Fitzgerald et al., 2018; Knight et al., 2013).

Another important aspect of inequality related to emissions is ecologically unequal exchange, a perspective that cuts across multiple social science disciplines, including anthropology, geography, and sociology. Unequal international exchange is the assertion of asymmetrical power relationships between more‐developed and less‐developed countries, as the former gain disproportionate advantages at the expense of the latter through trade patterns and global production networks. Ecologically unequal exchange refers to the environmentally damaging removal of energy and other natural‐resource assets from and the externalization of environmentally damaging production and disposal activities to less‐developed countries. Research in this tradition indicates that asymmetrical trade relationships and global production network characteristics contribute to the growth of energy use, production‐based carbon emissions and deforestation within developing nations (Bunker, 1984; Feng, Hubacek, & Yu, 2014; Givens, 2018; Hornborg & Martinez‐Alier, 2016; Huang, 2018; Jorgenson, 2006, 2012; Prell et al., 2015; Roberts & Parks, 2007). In a related vein, research on the environmental impacts of militarization (e.g., Bradford & Stoner, 2017; Clark et al., 2010), which is discussed in greater detail above, suggests that nations with larger and more technologically advanced militaries are more able to secure and maintain access to greater amounts of fossil fuels and other natural resources from different regions of the world, further leading to increased carbon emissions (Kentor, 2000; Tilly, 1992).

### 2.3 Demographic factors

The size and growth of the human population are well established as major drivers of environmental change, including carbon emissions, and much social science research provides empirical evidence supporting these claims (Burns et al., 1997; Dietz & Rosa, 1994, 1997; Jorgenson & Clark, 2010, 2013; Rosa, York, & Dietz, 2004; York, 2007; York, Rosa, & Dietz, 2003). However, the complex environmental effects of population growth, combined with other demographic factors, are less often documented. While population growth in poor nations, which tends to be higher than in rich nations, contributes to rising energy consumption and emissions, research in geography and sociology suggests that such growth threatens global climate stability less than wealthy nations' consumption practices do (Hubacek, Baiocchi, Feng, & Patwardhan, 2017; Jorgenson & Clark, 2013).

Beyond population size and growth, other demographic characteristics with important implications for emissions include age distribution, number of households, and average household size in a given population (Adua, York, & Schuelke‐Leech, 2016). Energy use and emissions tend to be higher when a larger share of the population is working aged (York, 2007). In developed countries, with larger aging populations, low fertility helps to suppress emissions, but the changing age structure only modestly limits emissions, at least in the short term (York, 2007). In some contexts, the number of households is a more important driver of environmental impacts than is the number of people (Liu, Daily, Ehrlich, & Luck, 2003; York & Rosa, 2012). Household size is declining in affluent nations, which leads to increases in energy consumption and carbon emissions (Weber & Matthews, 2008). Average household size also has begun to decline in rapidly developing countries as well (Leichenko & Solecki, 2005).

### 2.4 Land‐use transformation

Anthropologists, archeologists, geographers, and sociologists have demonstrated that land‐use transformation is an underlying cause of anthropogenic climate change. Some evidence to support this conclusion is derived from the long, continuous record of human‐induced changes. Land‐use transformation results from contextual and proximate causes. Contextual causes include a range of international market and institutional arrangements. Proximate causes are human activities that more directly contribute to emissions.

Archeologists demonstrate that such alterations have a long time span, from the Holocene's beginning (>10,000 years ago) and extending through the era of widespread agriculture, especially since about 7,000 years ago (d'Alpoim Guedes, Crabtree, Bocinsky, & Kohler, 2016; Erlandson & Braje, 2013; Ruddiman, 2005; Ruddiman & Ellis, 2009; Smith & Zeder, 2013). Both land‐use change and related biomass burning are important drivers of climate change in contemporary contexts; in particular, the agriculture, forestry, and other land‐use sector contributes to about 25% of net anthropogenic emissions, mainly from deforestation, agricultural soil‐ and nutrient‐management practices, and livestock (IPCC, 2014b).

Research in geography, anthropology, and sociology focuses on how interrelationships among national politics, international treaties, stratification, regions and scales combine to impact land use and land cover change at the district or municipal level (Smith et al., 2014). Proximate causes relate to a variety of household, community, and local infrastructural conditions (Rudel, 2005; Seto, Solecki, & Griffith, 2016; Turner, Moss, & Skole, 1993). Social scientists commonly study land transformation in rural domains, including the tropics, where they address the social and institutional processes of deforestation (Rudel, 2005). As important, their analyses of urban, suburban, and exurban land‐use and land‐cover change are critical for understanding urban residents' resource‐consumption patterns and associated greenhouse gas emissions (Leichenko & Solecki, 2005; Marcotullio et al., 2014; Romero‐Lankao et al., 2014; Rudel, 2009).

Landscape changes are also connected to large‐scale capital investments, including hydroelectric dam construction, large‐scale irrigation, and wetland drainage that permanently change local ecosystems. For example, Brazil's history of highway and hydro‐electric dam infrastructure development in the Amazon demonstrates how investments can lead to unanticipated and unsustainable population booms. These booms not only lead to challenges in human wellbeing through a lack of services, economic inequalities and loss of livelihood, but also related ecological challenges like deforestation, with attendant climate consequences (Fearnside, 1999; Moran, 2016; Richter et al., 2010; Walker, Moran, & Anselin, 2000). National governments often play active roles in development that results in deforestation, while local growth coalitions press for road building and development, even when national governments pull back from deforestation‐causing activities (Rudel, 2009).

## 3 MITIGATION AND ADAPTATION

Human responses to the risks and impacts of climate change largely fit into two categories: mitigation and adaptation (IPCC, 2014a, 2014b, 2014c). “Mitigation” refers to a human intervention to reduce the sources or enhance the sinks of carbon and other GHG (IPCC, 2014b). “Adaptation” refers to adjustments in natural or human systems in response to actual or expected climatic stimuli or their effects; such adjustments moderate harm or exploit beneficial opportunities (IPCC, 2014a). Both mitigation and adaptation occur at various spatial and temporal scales, using approaches that apply technological, economic, institutional, regulatory, ecosystem‐based, informational, and social factors (Carmin et al., 2015; Rosenzweig et al., 2018). In addition, mitigation and adaptation decisions are subject to path‐dependency, meaning current options are often constrained by the outcomes of past decisions (Ehrhardt‐Martinez, Rudel, Norgaard, & Broadbent, 2015). While the drivers of increasing atmospheric concentrations of GHG are largely international and global, the effects of contemporary climate change are experienced locally (Miller Hesed & Paolisso, 2015). This section discusses how approaches to mitigating and adapting to climate change are influenced by both long‐term and near‐term social processes as well as relationships between various actors.

### 3.1 Temporal contexts

By looking at long‐term changes from the past, archeology demonstrates that similar outcomes occurred in different areas that were affected by local climate change patterns (Redman, 1999). For example, the Long Term Vulnerability and Transformations Project based at Arizona State University, in collaboration with the North Atlantic Biocultural Organization, compares multiple societies' responses to sudden impacts of climate change in the thirteenth through fifteenth centuries CE. While the environment and societies were radically different, cases of successful adaptation had common underlying structural patterns. Although researchers have also identified painful transitions and full‐scale societal collapse, common successful adaptations include balancing population size against available resources, having a diverse portfolio of food and other choices, social networks that reduce risk, storage systems, mobility and migration, equal access to resources, and reduction of barriers to resources (Nelson et al., 2016). Historically, resistance to adopting tools from other cultures and over‐commitment to forms of fixed infrastructure, such as irrigation, have often led to adverse path dependency. Societal collapse is known to be associated with inflexible or out‐of‐phase management responses and the depletion of the social capital that legitimizes collective responses. Research by Chase and Scarborough (2014) suggests that collapse generally takes place well before total resource depletion and should therefore be understood as a management failure.

As it provides temporal context and shows longer term pathways, archeology also offers insights that can articulate with the shorter temporal scales that other social sciences usually consider. Historical ecological research, by combining archeology and history with other environmental social sciences and humanities, local and traditional knowledge, paleoecology, and the perspectives of modern resource managers, offers a broad framework for understanding deep time perspectives on human responses to and effects on climate change (Armstrong et al., 2017; Balée, 2006; Balée & Erickson, 2006; Braje, 2015; Braje & Rick, 2013; Burgi, 2011; Costanza et al., 2012; Egan & Howell, 2001; Hicks et al., 2016; Jackson & Hobbs, 2009; Meyer & Crumley, 2011; Rick & Lockwood, 2013).

The Resilience Alliance (2010) uses adaptive‐management strategies that draw upon long‐term perspectives developed through archeology. The Resilience Alliance and other interdisciplinary networks of scientists and practitioners work on not only improving response to sudden (often catastrophic) threshold crossing events but also identifying warning signals that such thresholds are being approached (i.e., forecasting tipping points, while there may still be time to mitigate and adapt). Threshold crossings are normally a complex mix of environmental and social factors, and developing a wider spectrum of such “red flag” variables can alert managers to oncoming transformations. For example, Streeter, Dugmore, Lawson, Erlendsson, and Edwards (2015) combine social and environmental variables and innovative use of volcanic tephra (ash) horizons in Iceland to mark human impacts on the Icelandic environment.

One component affecting this is the problem of Shifting Baselines, in which successive generations of resource managers perceive their current conditions as a natural baseline without recognizing longer term trends and patterns of simplification and degradation (Olson, 2002). This problem is well‐documented in fisheries and marine resource management (Campbell, Gray, Hazen, & Shackeroff, 2009; Pauly, 1995), but extends across terrestrial situations as well. For example, Engelhard, Righton, and Pinnegar (2014) have noted in an analysis of 100 years of North Sea cod distribution that both climate change and fishing pressure impact fish distributions.

### 3.2 Governance

In the area of governance and policy, the role and structure of international environmental agreements have been examined through multiple social science perspectives.

World Society Theory, a neo‐institutional tradition within sociology, highlights the role of global institutional structures in influencing social change and environmental outcomes (Meyer, Boli, Thomas, & Ramirez, 1997). World Society Theory argues that nation‐states are socially constructed actors embedded in a transnational system of structures, agents, and norms that legitimate and encourage some actions and not others. A central actor within world society theory is the International Nongovernmental Organization (INGO). Environmental INGOs are theorized to both reflect and carry forth the content of world society to nation‐states and subnational actors (Longhofer & Schofer, 2010). Studies in this tradition have found that ties to the pro‐environmental world society (a stronger presence of environmental INGOs) are associated with modest reductions in national‐level carbon emissions (Givens, 2017; Hironaka, 2014; Schofer & Hironaka, 2005; Shandra, London, Whooley, & Williamson, 2004). Research in this tradition also indicates that the effect of economic growth on carbon emissions has moderately decreased in magnitude through time in nations that are most central in the global network of environmental INGOs (Longhofer & Jorgenson, 2017). In other words, world society integration can help facilitate a decoupling between economic development and emissions.

Research in political sociology also shows the importance of governance structure. A recent study employs multilevel modeling techniques to analyze carbon emissions from fossil‐fuel power plants in the 25 post‐Soviet transition nations in Central and Eastern Europe and Eurasia (Jorgenson, Longhofer, Grant, Sie, & Giedraitis, 2017). Various plant‐level factors are associated with higher emissions, including coal as the primary fuel source, plant size and age, capacity utilization rate, and heat rate. Regarding governance, results indicate that plant‐level emissions are lower, on average, in the transition nations that joined the European Union (EU), whose market reforms and environmental directives are quite relevant for emissions reductions. These negative associations between plant‐level emissions and EU accession are larger for the post‐Soviet nations that joined the EU earlier relative to those that joined more recently.

In the United States as well, environmental regulations can lead to reductions in carbon emissions from fossil‐fuel burning power plants. Analyzing plant‐level and state‐level data with multilevel modeling techniques, sociologists Grant, Bergstrand, and Running (2014) assess state policy effects on individual power plants' emissions. Both direct strategies, such as emission caps and targets, and indirect strategies, such as public benefit funds, lower plants' emissions and thus can be viable building blocks in a federal climate regime. Other recent research, using longitudinal data from all 50 U.S. states, indicates that the effects of population and affluence on state‐level carbon emissions are substantially moderated by congressional representatives' pro‐environmental voting (Dietz, Frank, Whitley, Kelly, & Kelly, 2015). Political‐institutional factors, such studies show, can ameliorate the environmental effects of economic and demographic factors.

Other bodies of research in sociology and geography have pointed to subnational opportunities to fill what has been called a climate “policy void” in U.S. politics (Fisher, 2013; Jones, 1991; Krane, 2007; Rabe, 2007; Shwom, 2011). In some cases, this work encourages the multi‐level governance of climate change, which crosses scales and frequently involves a broader range of policy actors in the decision‐making process (Bulkeley, 2005; Galli & Fisher, 2016). Research in this area also maps out how networks of policy elites are engaging in the climate debate (Fisher et al., 2018; Fisher, Leifeld, & Iwaki, 2013; Fisher, Waggle, & Leifeld, 2013). Similar claims are made in the social science literature on polycentric governance (Cole, 2015; Dorsch & Flachsland, 2017; Gillard, Gouldson, Paavola, & Van Alstine, 2017; Hsu, Weinfurter, & Xu, 2017; Ostrom, 2014; Spreng, Sovacool, & Spreng, 2016), with some focus on global climate politics since the Paris Agreement was signed in 2015 (Oberthür, 2016; Victor et al., 2017). Polycentricity refers to a form of governance with multiple centers of semiautonomous decision making. Scholars have argued that if decision‐making centers take each other into account in competitive and cooperative relationships and have recourse to conflict resolution mechanisms, they may be regarded as a polycentric governance system (Carlisle & Gruby, 2017).

Anthropologists have engaged political ecology theories to analyze the effectiveness of governance structures and approaches. Focusing on resource management and the commons, such research has assessed the role of the state and private property with respect to tragedies of the commons where individuals in a shared‐resource context are posited to act for their own individual interests. This is common in smaller‐scale societies, but also found in developed nations where local control is embedded in a national framework (McCay & Acheson, 1987; Pinkerton, 2011).

Global mitigation policies, developed to reduce deforestation and increase carbon sequestration in the world's forests, include CDM (Clean Development Mechanism), REDD (Reducing Emissions from Deforestation in Developing Countries), and REDD+ (Reducing Emissions from Deforestation and Forest Degradation in Developing Countries). Globally, while REDD and REDD+ have enhanced stewardship and reduced land degradation, deforestation continues to increase in Indonesia, Malaysia, and parts of Africa and South America, and the discontinuities between top‐down policies with activities occurring at the project or community level can be seen in the implementation of such programs. Concerns have arisen about equity and the policies' effectiveness (Harlan, Pellow, & Roberts, 2015; Paladino & Fiske, 2017; Parks & Roberts, 2010). In an examination of 9 cases in Uganda, Nel (2015, 2017) finds that benefits are asymmetrical, and local people are often affected by “expulsion and marginalization”. However, research on the Khasi Hills Project in India indicates that community‐based forest management has the potential to provide an effective strategy for mitigating powerful drivers of deforestation, and can be even more successful if supported through internationally recognized and certified carbon projects, including both REDD+ and Afforestation and Reforestation (Poffenberger, 2017). The concept of REDD+ is evolving and one of the more promising approaches is “Jurisdictional REDD,” which spans different land‐use types across landscapes and with multiple stakeholders in a subnational jurisdiction (Fishbein & Lee, 2015). More broadly, this area of research indicates that global‐scale initiatives need to connect more effectively to local environmental and social contexts (Angelsen & Rudel, 2013; Fiske et al., 2014; Lansing, 2012; Leach & Scoones, 2015; Paladino & Fiske, 2017).

CDMs use carbon offsets to manage anthropogenic climate change, generally by harnessing technology or engineered solutions through large‐scale energy generation plants or chemical manufacturing facilities that use technology to capture carbon. Social science critiques have identified limitations of CDM programs and policies. For instance, capital flows from offsetting in the compliance market mirror distributional inequities of direct foreign investment: sub‐Saharan Africa attracts less than 2% of such investment, while China, Brazil, and India—the three largest recipients—together receive the bulk of the CDM investment (Bailey, Gouldson, & Newell, 2012). In addition, there are numerous unrealized goals, including generating carbon‐reducing activities and projects promoting cobenefits for more sustainable community‐level development (Bailey et al., 2012). Among other areas of concern addressed in the social science literature on CDMs are institutional structures, including the use of markets, and unintended incentives and consequences (Boyd et al., 2009; Boyd, Boykoff, & Newell, 2012; Boyd, Gutierrez, & Chang, 2007; Brown & Corbera, 2003; Finley‐Brook, 2016).

### 3.3 Technology

Energy use has evolved over millennia, and because its current concentration in fossil fuels is integral to economic growth, changing that concentration will likely be difficult within the structure of the contemporary world economy (Antonio & Clark, 2015; Chase‐Dunn, 1998; Clark & York, 2005; Hornborg, 2013; Rosa et al., 2015; Smil, 2010; Strauss, Rupp, & Love, 2013; Urry, 2016; White, 2016). Technological options often provide short‐term fixes but often have long‐term, unanticipated, impacts. For example, increasing energy efficiency through technological innovations is often assumed to be an effective strategy for reducing energy consumption and associated greenhouse gas emissions. Efficiency lowers the price of energy and related services, however, so it may increase demand for them and thereby cause total emissions to rise—a point that William Stanley Jevons first argued in the nineteenth century (York & McGee, 2016), and similar to what is commonly referred to as “rebound effects.”

Sociologists Grant, Jorgenson, and Longhofer (2016) analyze a dataset containing nearly every fossil fuel power plant in the world with multilevel modeling techniques to determine whether the impact of efficiency on emissions varies by plants' age, size, and location in global economic and normative systems. Their findings indicate that each of these factors has a significant interaction with efficiency and thus shapes environmentally destructive rebound effects. Related research finds that the dirtiest 5% of fossil fuel power plants in nations throughout the world are disproportionately responsible for large shares of their sectors' total emissions. If these plants continued generating the same amount of electricity but met particular intensity targets through enhanced efficiencies or through other means, the world's total electricity‐based carbon emissions could be reduced by as much as 40% (Grant, Jorgenson, & Longhofer, 2013; Jorgenson, Longhofer, & Grant, 2016).

In an effort to effectively identify hyper‐polluting plants throughout the world that are disproportionately responsible for the electricity generation sector's total emissions, Grant, Jorgenson, and Longhofer (2018) employ qualitative comparative analysis (QCA) techniques to analyze the conjoint effects of global, political, and organizational conditions on fossil‐fueled plants' carbon emissions. QCA treats cases as combinations of attributes and uses Boolean algebra to derive expressions of combinations associated with an outcome. Such a technique is well suited to evaluate higher‐order interactions and determine which of several possible combinations of factors are most relevant for an outcome. Their findings reveal that hyper‐polluters' emission rates are a function of four distinct causal recipes, which they label coercive, quiescent, expropriative, and inertial configurations, and these same sets of conditions also increase plants' emission levels (Grant et al., 2018). Coercive and quiescent configurations enhance plants' ability to externalize their carbon emissions by neutralizing and manipulating potential sources of resistance, whereas expropriative and inertial configurations inhibit plants' ability to curb emissions by subjecting them to opportunistic behavior and forces of inertia.

An important example of the unanticipated consequences of technological change is the role of renewable energy: it is not necessarily the case that adding renewable energy sources without structural changes to the economy will reduce fossil fuel use. Sociological research by York (2012b) indicates that in nations around the world since 1960, growth in nonfossil fuel sources only minimally displaced fossil fuel use (controlling for economic growth, population growth, and other factors)—that is, nonfossil energy sources were largely added to, rather than in place of, fossil energy sources (see also Greiner, York, & McGee, 2018; Thombs, 2018b). This finding, although superficially surprising, fits with a long tradition of research in technology studies which finds that technologies often have unanticipated consequences, due to interactions with social, economic, and political forces. Other sociological research suggests that interactions between the increasing use of renewable energy sources and economic growth may also lead to a tighter coupling of GDP to carbon emissions because renewables are more likely to be used to replace nuclear power than to replace fossil fuels, thus maintaining the dominance of fossil fuels as the base load electricity source (Thombs, 2017; York, 2016, 2017; York & McGee, 2017).

However, it may be feasible to achieve near‐term reductions in carbon emissions by the adoption and use of readily available technologies, according to a study of U.S. homes and nonbusiness travel (Dietz et al., 2009). Dietz and colleagues use data on the most‐effective documented interventions to estimate the plasticity (which measures the ease and speed of change) of 17 household action types in behaviorally distinct categories. These interventions involved several policy tools and strong social marketing but not new regulatory measures. Within 10 years, they estimate, nationwide implementation could save 123 million metric tons of carbon with little or no reduction in household well‐being (see also Shwom & Lorenzen, 2012).

### 3.4 Decarbonization

Research on decarbonization has focused on evaluating the feasibility, technology pathways, and costs of near‐term and long‐term mitigation scenarios. Many scenario studies of long‐term climate stabilization use modeling frameworks with representation of the global energy economy (Fawcett et al., 2015; IPCC, 2007, 2014b; Riahi, Gruebler, & Nakicenovic, 2007), while other scenario modeling research focuses more narrowly, on such cases as the U.S. economy (Paltsev, Reilly, Jacoby, & Morris, 2009; Risky Business Project, 2016) and energy sectors (McCollum & Yang, 2009). Some research in geography and related fields separates sector, region, city, and time periods to address infrastructure changes, technology deployment, sectoral investment, and associated behavioral patterns of low‐carbon transitions (Bataille, Waisman, Colombier, Segafredo, & Williams, 2016; C40 and Arup, 2016; Mileva, Johnston, Nelson, & Kammen, 2016; Solecki et al., 2018). Scenario‐based projections suggest potential opportunities for decoupling economic growth from global‐ and local‐scale carbon emissions (Loo & Banister, 2016; Shen & Sun, 2016), while research at municipal and neighborhood levels defines differential emissions rates under different socioeconomic conditions and ecosystem regimes (Hardiman et al., 2017; Liu, Ma, & Chai, 2017).

However, current knowledge about transitions to a low‐carbon economy and deep decarbonization is limited by a lack of empirical evidence: there are no known cases in which societies or nations have deliberately and systematically deeply decarbonized. Nonetheless, the conditions and prospects of a socially feasible decarbonization transition are increasingly addressed in social science literature. The issues considered include governance capacity; social, political and institutional adjustments across different scales; dimensions of well‐being; attitudes and behavior; benefits; innovation diffusion; equity and justice; conditions of data; information limitations and uncertainty (Betsill & Bulkeley, 2006; Busby & Shidore, 2017; Byravan et al., 2017; Geels, Sovacool, Schwanen, & Sorrell, 2017). Cobenefits of climate change mitigation are also examined (Ibrahim, 2017), and a growing literature considers such applications in cities and urban contexts (Bulkeley, Edwards, & Fuller, 2014; Hodson, Geels, & Mcmeekin, 2017; Hughes, 2017; Luque, Edwards, & Lalande, 2013; McGuirk, Bulkeley, & Dowling, 2016).

## 4 CONCLUSION

This article has summarized bodies of recent research in anthropology, archeology, geography, and sociology on the drivers of climate change, with a focus on the anthropogenic drivers of carbon emissions and factors that influence the effectiveness of mitigation and adaptation strategies. As a whole, the research reviewed from these social science disciplines highlights that among the key human factors contributing to climate change are the roles of and connections among economic conditions and development; demographic growth and changes; power, social stratification and inequality; technology; infrastructure; and land‐use change. These factors' near‐ and long‐term dynamic interactions across spatial scales and institutional contexts shape the pathways and options for mitigation and adaptation.

Economic activities, and associated growth in income and consumption are major drivers of carbon emissions. Power, social stratification and forms of inequality are often key factors that shape outcomes, including carbon emissions at national and subnational levels. Analyses at the micro‐level, such as the household, and in particular spaces, such as urban areas, emphasize that sociocultural contexts are important for understanding consumption as a driver. Population growth is a major driver of climate change, but not all humans contribute equally to carbon emissions.

Land use and land transformation are important drivers of climate change because they result from complex interactions on multiple levels. Significant aspects include global treaties, global and local economic forces, national policies and politics, urban–rural relationships, household behaviors, and local infrastructure. Along with exploring this complexity, the social sciences offer alternative adaptation and mitigation strategies that take into account historical ecology and different temporal‐scale relationships between the natural world and the social world.

Long‐term perspectives on drivers of climate change and human pathways help in comprehending thresholds and tipping‐points and in building planning scenarios. Understanding the current impact of past human activities and the long‐term evolutionary processes that drive human behaviors are critical not only for understanding the drivers of climate change but also for creating mitigation and adaptation efforts. Effective global‐scale policies and initiatives must connect to regional and local conditions and social contexts.

Decarbonization requires dramatic changes in energy systems and policies. Consideration of how policies influence not only the availability of low‐ and noncarbon energy technologies but also total energy production and consumption can lead to more sustainable outcomes. Technologies have unintended and unanticipated consequences due to interactions with social, economic, and political forces. In order to effectively reduce carbon emissions, structural changes, such as reducing income inequality, increasing sustainable consumption, and implementing effective regulatory mechanisms, are all necessary.

The areas of social science literature reviewed in this article point to multiple avenues for future data collection and research. First, there is a significant need to fill data gaps at household, community, and other local levels on drivers as well as mitigation‐ and adaptation‐related issues. Second, we need to increase our understanding of consumer demands, choices, and commodity use, all of which will help target areas in which to reduce emissions. Third, there remains a need to integrate more fully knowledge of physical and social systems, both for understanding driver‐related pathways and for creating successful adaptation and mitigation opportunities.

A fourth important area to address is developing clearer pathways on all levels for moving historical data and knowledge into praxis. Important considerations are renewable energy and jobs production, household and industry subsidies for renewable energy adoption, alternative models for economic growth, and how to decarbonize while ensuring ecologically sustainable and socially equitable development. Fifth, there is a need for much more systematic cross‐regional comparisons of cases involving long‐term human environmental dynamics, which could aid in generalizing about long‐term lessons.

Sixth, there is a need for improvements in forecasting thresholds and tipping points of both social and natural systems. These improved predictions are needed for societal responses to sudden, often catastrophic threshold‐crossing events and warnings of their approach while there is still time to mitigate and plan for adaptation. A seventh area for future attention is correcting assumptions about shifting baselines. Problems arise when successive generations of resource managers and researchers perceive current conditions as a natural baseline against which to evaluate future events, rather than recognize long‐term trends and patterns of simplification and degradation that may have occurred in prior decades, centuries, or millennia.

Eighth, traditional carbon emissions accounting (e.g., territorial or production‐based), does not measure the extent to which environmentally harmful production is outsourced abroad. Consumption‐based accounting, by shifting system boundaries, facilitates tracking carbon emissions along regional and global supply chains, and reallocates those emissions to the final consumer. Thus, research that focuses on international inequality perspectives, such as ecologically unequal exchange, would do well to analyze both consumption‐based and production‐based measures of emissions.

Finally, research questions must focus more directly on the relationship between decarbonization and economic growth. With this, studies are needed of the social‐structural, institutional, technological, and behavioral conditions that would ensure socially feasible decarbonization transitions, especially given the scale of carbon sequestration required and the associated impacts on land use and food prices, and the ways in which a low‐carbon economy would affect individual well‐being and social equity.

## CONFLICT OF INTEREST

The authors have declared no conflicts of interest for this article.

## RELATED WIREs ARTICLES

Human well‐being and climate change mitigation
